# Accelerometric Classification of Resting and Postural Tremor Amplitude

**DOI:** 10.3390/s23208621

**Published:** 2023-10-21

**Authors:** Christina van der Linden, Thea Berger, Gregor A. Brandt, Joshua N. Strelow, Hannah Jergas, Juan Carlos Baldermann, Veerle Visser-Vandewalle, Gereon R. Fink, Michael T. Barbe, Jan Niklas Petry-Schmelzer, Till A. Dembek

**Affiliations:** 1 Department of Neurology, University Hospital Cologne and Faculty of Medicine, University of Cologne, 50937 Cologne, Germany; christina.van-der-linden@uk-koeln.de (C.v.d.L.); jan.petry-schmelzer@uk-koeln.de (J.N.P.-S.); 2 Department of Stereotactic and Functional Neurosurgery, University Hospital Cologne and Faculty of Medicine, University of Cologne, 50937 Cologne, Germany; 3 Department of Psychiatry and Psychotherapy, University Hospital Cologne and Faculty of Medicine, University of Cologne, 50937 Cologne, Germany; 4 Institute of Neuroscience and Medicine (INM-3), Forschungszentrum Jülich, 52428 Jülich, Germany

**Keywords:** tremor, accelerometry, Parkinson’s Disease, essential tremor, wearables

## Abstract

Clinical rating scales for tremors have significant limitations due to low resolution, high rater dependency, and lack of applicability in outpatient settings. Reliable, quantitative approaches for assessing tremor severity are warranted, especially evaluating treatment effects, e.g., of deep brain stimulation (DBS). We aimed to investigate how different accelerometry metrics can objectively classify tremor amplitude of Essential Tremor (ET) and tremor in Parkinson’s Disease (PD). We assessed 860 resting and postural tremor trials in 16 patients with ET and 25 patients with PD under different DBS settings. Clinical ratings were compared to different metrics, based on either spectral components in the tremorband or pure acceleration, derived from simultaneous triaxial accelerometry captured at the index finger and wrist. Nonlinear regression was applied to a training dataset to determine the relationship between accelerometry and clinical ratings, which was then evaluated in a holdout dataset. All of the investigated accelerometry metrics could predict clinical tremor ratings with a high concordance (>70%) and substantial interrater reliability (Cohen’s weighted Kappa > 0.7) in out-of-sample data. Finger-worn accelerometry performed slightly better than wrist-worn accelerometry. We conclude that triaxial accelerometry reliably quantifies resting and postural tremor amplitude in ET and PD patients. A full release of our dataset and software allows for implementation, development, training, and validation of novel methods.

## 1. Introduction

Tremor is the main symptom of Essential Tremor (ET) and is also present in most patients with Parkinson’s Disease (PD). Rating scales such as the Fahn–Tolosa–Marin Tremor Rating Scale (FTM-TRS) [1], the Essential Tremor Rating Assessment Scale (TETRAS) [2], and the MDS-UPDRS [3] are used for diagnostic purposes and to assess the treatment effects of therapies like deep brain stimulation (DBS) in clinical settings as well as in the context of clinical studies. An ordinal scale (0–4 points) is applied to assess tremor severity in different tasks–resting, postural and kinetic tremor. While these scales are broadly used and useful for assessing tremor, several limitations exist [4]. Firstly, ordinal rating scales have a low resolution [5], so there are difficulties depicting small differences in tremor severity. This can be particularly relevant in the context of complex tremor therapies such as deep brain stimulation (DBS), where discrete changes in tremor amplitude achieved by optimizing stimulation parameters can be highly relevant for patients in their daily lives. Additionally, following Weber’s law of psychophysics, the smallest discernible change in tremor tends to depend on the initial tremor amplitude in clinical rating scales [5], and there are relevant ceiling effects [4]. Furthermore, moderate interrater reliability [3,6,7,8,9], differences due to rater experience [8] and within-subject reliability [10] limit the use of clinical rating scales in patient care, especially in monitoring patients over a longer period of time with multiple clinicians involved.

Accelerometry might be a feasible and reliable technique to accurately and objectively measure tremor severity and overcome some limitations [11,12,13,14]. Accelerometry results can be correlated with clinical ratings using rating scales based on a nonlinear relationship [4,5]. In a previous study comparing intraoperative clinical PD tremor ratings and accelerometry-predicted ratings, a substantial agreement was found for resting and postural tremors according to Cohen’s weighted Kappa [15].

Accelerometry has been applied in several clinical studies to assess the treatment effects of medication [16], DBS [17] or for intraoperative evaluation of tremor severity during thalamotomy surgery [18]. Recently, wearable accelerometers [12,19] and other portable devices such as smartwatches [20] and smartphones [21,22] have been used to detect and evaluate tremors with high precision [23]. Certain devices and solutions have even gained regulatory approval to monitor tremor in PD patients continuously [20].

However, the applied methods vary among the published approaches analyzing tremors, especially regarding preprocessing steps such as filtering or algorithms estimating spectral density and regarding the different parameters depicting tremor severity [11,12,15,16,24]. Most approaches focus on spectral metrics such as peak or total power of acceleration [12] or the area under the power spectral density curve [15], while others take tremor frequency into account [11,12,18]. Further, scripts and pipelines are often not publicly available or generally applicable due to differences in, e.g., measurement characteristics [12,15]. Furthermore, in most studies proposing accelerometry-based algorithms for tremor analysis, sample sizes were small, and algorithms were not validated in out-of-sample data [12,25].

The aim of this study was to investigate how accelerometry measurements can best quantify tremor amplitude of ET and Parkinsonian tremor. In a large dataset, we aimed to compare different accelerometry parameters collected at the wrist and fingers concerning their ability to predict clinical tremor severity in out-of-sample data. We aimed to investigate accelerometry’s accuracy in classifying tremors and its comparability to the accuracy of clinical rating scales. We curate and release our full dataset and methodology for the explicit purpose that other groups can build on our solutions and use this dataset to validate their own approaches externally.

## 2. Materials and Methods

### 2.1. Dataset

We conducted tremor analysis in 41 patients treated with DBS for ET (n = 16) or PD (n = 25) at University Hospital Cologne (13 female/28 male), mean age 60.1 (±9.9) years. All but one patient were examined without tremor suppressing or dopaminergic medication. Patients were then investigated in the DBS OFF state, and upper limbs were only further investigated when they had clinically significant baseline tremor (for PD resting tremor ≥ 2/4 MDS-UPDRS Item 3.17, for ET postural or intention tremor ≥ 2/4 FTM-TRS Item 5 or 6) in the medication OFF and stimulation OFF state. Patients were then repeatedly examined under different DBS parameter settings, varying contact selection and stimulation amplitude as part of an ongoing clinical trial, resulting in up to 17 tremor assessments per upper limb. After excluding missing data, the dataset included 860 trials in 54 upper limbs (ET n = 27; PD n = 27) for both postural and resting tremor. We randomly split this dataset into training data with data from n = 15 upper limbs each for ET and PD (479 trials) and validation data (n = 12 upper limbs each, 381 trials). This analysis is part of an ongoing trial registered with the German Clinical Trials Register (DRKS00026596). The trial was approved by the local ethical review board (vote: 21-1441) and conducted under the Declaration of Helsinki and the guidelines for good clinical practice. Data were collected at the Department of Neurology of the University Hospital Cologne. All patients provided written informed consent prior to study participation.

### 2.2. Tremor Tasks

In each trial, we assessed resting tremor and postural tremor following the instructions of MDS-UPDRS III Items 3.15 and 3.17 [3]. If necessary, the position of the hand or arm was slightly modified to provoke the largest tremor amplitude possible. Patients were given standardized video-based instructions for each trial. Each position was maintained for 20 s, with each trial’s start and end point indicated by an acoustic signal. Corresponding timestamps were recorded using the PsychToolbox Version 3.0.19 [26,27] extension in MATLAB.

### 2.3. Clinical Rating

Tremor was rated by a movement disorders clinician (C.v.d.L.) experienced in assessing tremor according to tremor amplitude cutoffs provided by MDS-UPDRS Items 3.15–3.17 (0 (normal)—no tremor; 1 (slight)—tremor is present but tremor amplitude < 1 cm; 2 (mild)—tremor amplitude 1–3 cm; 3 (moderate)—tremor amplitude 3–10 cm; 4 (severe)—tremor amplitude > 10 cm) [3]. If the tremor amplitude fluctuated, the maximum amplitude seen during the trial was rated as suggested by the MDS-UPDRS instructions. For each task, only the 20 s trial duration was considered, and tremor occurring outside this period was ignored. Additionally, 100 trials (n = 47 StimOFF and n = 53 randomly selected stimulation settings) were rated from video recordings by a second experienced movement disorders clinician (G.B.) for validation. Both raters were unaware of the accelerometry results and the other rater’s assessment during clinical rating.

### 2.4. Accelerometry

All trials were simultaneously recorded using two different accelerometry devices (Supplementary Figure S1). We attached a BrainProducts (BrainProducts GmbH, Gilching, Germany) triaxial accelerometer to the proximal dorsum of the index finger (FingerACC; Sampling rate 2500 Hz; ±2 g acceleration sensing range; <0.001 g resolution). Furthermore, patients wore a wristwatch accelerometer (GENEActiv© Original, Activinsights^TM^, Kimbolton, UK) with previous applications for tremor evaluation in ET [12] and PD [28] (WristACC; sampling rate 100 Hz; ±8 g acceleration sensing range; 0.004 g resolution). Data from both devices were converted from their respective data formats to MATLAB-compatible data using in-house scripts.

### 2.5. Preprocessing

Both FingerACC and WristACC data were segmented into trials according to the timestamps provided by our task. The first 2 s of each trial were discarded to remove any voluntary movement at the beginning of the task. While WristACC data from the GENEActiv device were already provided in [g], FingerACC data from the BrainProducts device were converted from [µV] to [g] according to the device specifications. We then renamed the axes of both datasets to a common system regarding the center of the extended hand, in which the X-axis pointed towards the thumb, the Y-axis pointed towards the fingertips, and the Z-axis pointed upwards from the back of the hand. We applied a 6th-order Butterworth bandpass filter from 1 Hz to 40 Hz, as described by Zach et al. [16], to exclude slow signal shifts or fast oscillations unlikely to be associated with the tremors.

### 2.6. Spectral Metrics of Tremor

Accelerometry data filtered from 1 to 40 Hz were used for further spectral analyses. For each trial, we calculated Welch’s power spectral density (PSD) estimate with a window width of 1 s and a 50% window overlap for frequencies in steps of 0.5 Hz (MATLAB: pwelch()). We calculated two different parameters for tremor amplitude from the resulting power spectrum.

**Peak Power.** First, as in other publications [12,16], we calculated the amplitude of the largest peak (Peak Power, [g^2^/Hz]) within the power spectral density between 3 Hz and 12 Hz as this covers the typical frequency range of resting and postural tremor in ET (4–12 Hz) [29] and PD (3–7 Hz) [11,30]. If there was no peak, the peak amplitude was set to zero. If there was more than one peak, the peak with the larger amplitude was chosen.

**AUC Power.** Second, as described in Smid et al. [15], we calculated the area under the power spectral density curve (AUC Power, [g^2^/Hz^2^]) between 3 Hz and 12 Hz, covering the frequency range of both ET and PD tremor.

### 2.7. Acceleration Metrics of Tremor

Besides the spectral analyses, we performed analyses of the acceleration data. Since we were only interested in tremor-related acceleration, data for these analyses were filtered using a 6th-order Butterworth filter with a bandpass from 3 to 12 Hz.

**Mean Envelope.** We calculated the Mean Envelope by identifying local maxima and minima within the filtered acceleration data and then using a spline interpolation to calculate the envelope (MATLAB: envelope(‘peak’)). The total envelope was calculated by summating all envelope values and then averaged by dividing by the number of samples (duration × sampling rate). Consecutively, the Mean Envelope in [g] is independent of both sampling rate and trial duration.

**Mean Acceleration.** As the simplest metric, Mean Acceleration was calculated by summating all absolute acceleration values within the filtered acceleration data. The resulting sum was then divided by the number of samples to obtain the Mean Acceleration in [g], which again is independent of the accelerometry device’s trial duration and sampling rate.

### 2.8. Relationship to Clinical Ratings

It is well known that the relationship between tremor amplitude and clinical rating scales of tremor amplitude is nonlinear [5]. Two major relationships have been previously proposed. The first is a logarithmic relationship according to the Weber–Fechner law [5,31], illustrated in Equation (1):

(1) $$TR=a\times \ln\left(T\right)+b$$

wherein *T* is the tremor amplitude, *TR* is the amplitude rating on a clinical scale, *a* is the slope, and *b* is the intercept of the linear relationship between the natural logarithm of *T* and *TR*. Alternatively, a relationship according to Steven’s power law has been proposed [5], illustrated in Equation (2):

(2) $$TR=a\times T^{c}+b$$

wherein *c* is the exponent of a power relationship between *T* and *TR*. Note that both equations (contrary to their original formulation) also contain an intercept term *b*, as Elble et al. proposed [5].

We performed separate nonlinear regression analyses for both relationships, and each of the different tremor analysis approaches explained above using the *fitnlm* function in MATLAB. Nonlinear regression analysis was performed with all data in the *training dataset* to determine the optimal model parameters for *a*, *b*, and *c*. The resulting optimal models were then applied to our *validation dataset* to predict the clinical tremor rating.

### 2.9. Statistical Analysis

**Figure 1:**
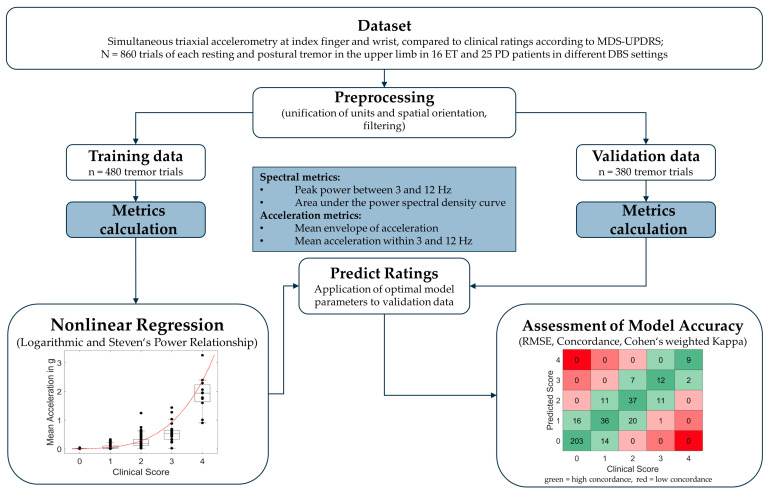
Overview of study methodology. ET = Essential Tremor; PD = Parkinson’s Disease; RMSE = root mean standard error.

To assess the accuracy of the respective models in predicting the clinical tremor rating from accelerometry measurements, we provide the root mean standard error (RMSE). While the predictions generated by our nonlinear models were continuous, we also generated predicted 0–4-point ratings by rounding model predictions to the next integer. These were then compared to the clinical ratings via weighted Cohen’s Kappa and the percentage Concordance of predicted and clinical ratings. We also report how often predicted ratings differed from clinical ratings by 1 point and how often they differed by > 1 point. For an overview of the study methodology, please see Figure 1.

### 2.10. Post hoc Comparison of ET and PD

In a post hoc analysis, we additionally investigated whether model accuracy was the same for both diseases by applying our models, which were trained on all subjects of the *training dataset*, separately on the subjects with ET or PD in our validation dataset. We also investigated whether predictions improved when training the models only on the subjects with the respective disease in our *training dataset*.

### 2.11. Technical Realization and Data Availability

All analyses were performed with MATLAB (R2022a, The MathWorks, Natick, MA, USA). The Open Science Framework (OSF) provides the full analysis pipeline. Additionally, we provide annotated scripts that can be used to calculate tremor metrics in new data, and all accelerometry data and clinical ratings are provided in an annotated dataset so that they may be used for validation purposes in other current or future approaches. All scripts and data are available under the creative commons license CC BY-SA 4.0 and can be found at: https://dx.doi.org/10.17605/OSF.IO/MP5HA (accessed on 17 September 2023). We include .csv files of all the data, MATLAB scripts to reproduce our analyses, and a tremor calculator script that allows us to calculate all the above-mentioned metrics and the respective model tremor scores for new data provided by the user.

## 3. Results

### 3.1. Training Data

Our training dataset consisted of n = 478 trials of resting tremor and postural tremor (n = 477 for WristACC). Tremor parameters were calculated, and nonlinear models were fitted for resting and postural tremor, WristACC and FingerACC, and log and power relationships, respectively.

### 3.2. Validation Data

Our validation dataset consisted of n = 380 trials of resting and postural tremor (n = 379 for FingerACC). Fitted models from our training dataset were applied to our validation dataset to predict tremor scores. Root mean squared errors (RMSE) when applying the trained models to our validation dataset typically lay around 0.5 points. Model parameters and accuracy of predicted scores versus clinical ratings are summarized in Table 1 for logarithmic relationships (Equation (1)) and Table 2 for Steven’s power relationships (Equation (2)).

**Figure 2:**
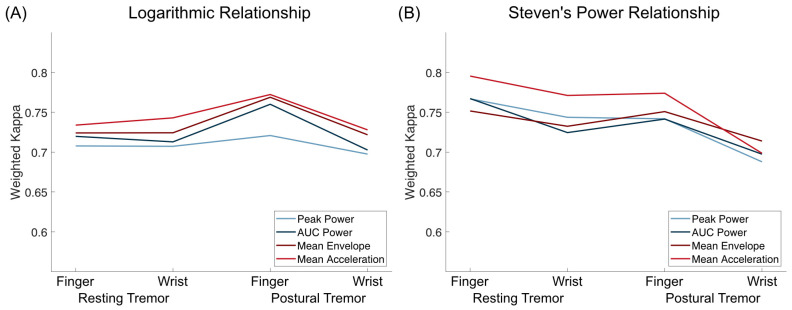
Weighted Kappa values for logarithmic relationships (**A**) and Steven’s power relationships (**B**) for both resting and postural tremor and finger and wrist accelerometry. Mean Acceleration was the tremor metric with the highest weighted kappa values for every setting except for postural tremor assessed by WristACC, assuming a Steven’s power relationship.

Overall, there was a high concordance and substantial interrater reliability between accelerometry-predicted and clinical ratings (see Table 1 and Table 2 and Figure 2). Modeling based on Steven’s power relationship provided slightly more accurate results than the models based on logarithmic relationships. The acceleration-based metrics performed slightly better than the frequency-based metrics, with Mean Acceleration demonstrating the highest weighted Kappa values for resting tremor (FingerACC κw = 0.795, WristACC κw = 0.771) and postural tremor and FingerACC (κw = 0.774). For postural tremor and WristACC, the Mean Envelope metric performed best with κw = 0.714 (only results from Steven’s power relationships are reported here, for logarithmic relationships, see Table 1 and Supplementary Table S1).

**Figure 3:**
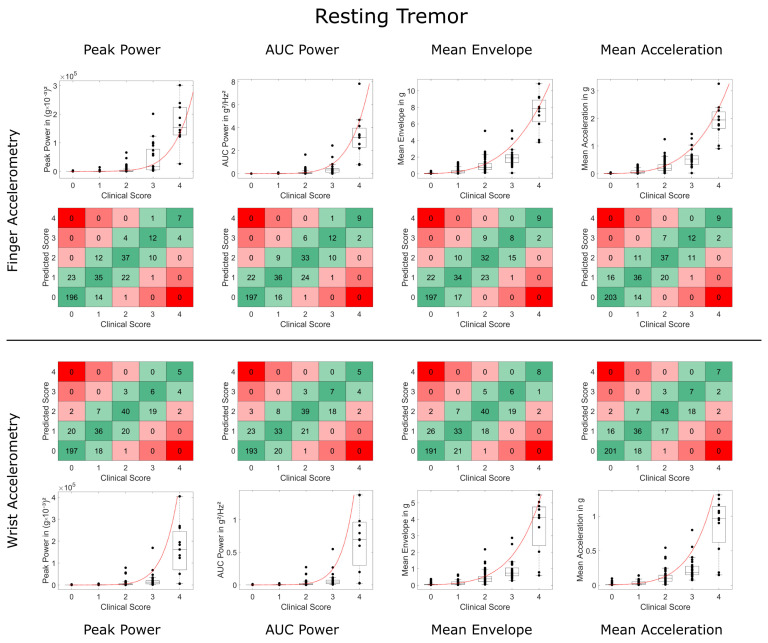
Comparison of accelerometry metrics and clinical scores for resting tremor. Data on finger and wrist accelerometry are shown in the two upper and lower rows, respectively. Boxplots and black data points depict the validation data and Steven’s power model trained on the training data (in red). Square plots depict deviations between the predicted tremor score and clinical tremor rating of more than 1 point in red (2 points deviation in light red, 3 and 4 points deviation in dark red). High agreement (no deviation) is highlighted in dark green, deviation of only 1 point in light green.

**Figure 4:**
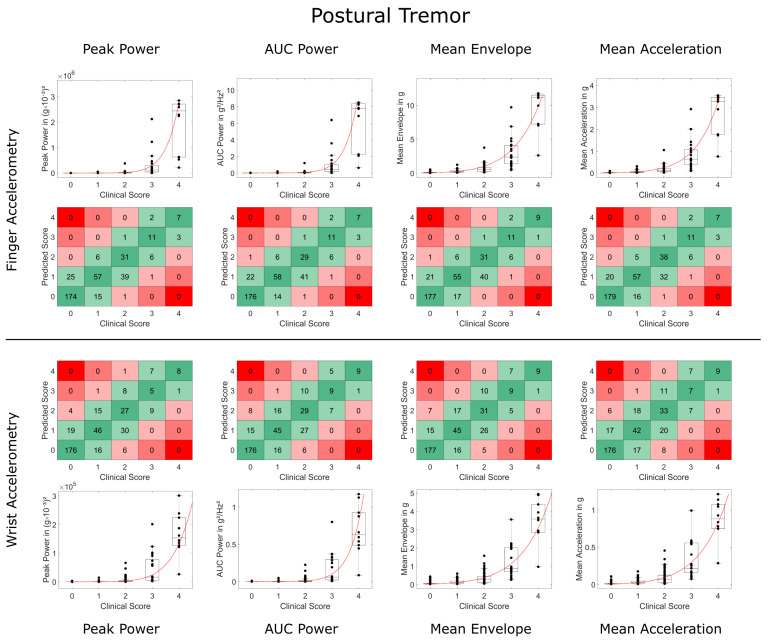
Comparison of accelerometry metrics and clinical scores for postural tremor. Data on finger and wrist accelerometry are shown in the two upper and lower rows, respectively. Boxplots depict the validation data and Steven’s power model trained on the training data (in red). Square plots and black data points depict deviations between the predicted tremor score and clinical tremor rating of more than 1 point in red (2 points deviation in light red, 3 and 4 points deviation in dark red). High agreement (no deviation) is highlighted in dark green, deviation of only 1 point in light green.

Weighted Kappa and concordance values were slightly but consistently lower for WristACC than FingerACC. In case predicted scores and clinical ratings differed, they typically deviated by 1 point, with more than 1 point deviations occurring in less than 1% of the conditions for most metrics (Table 1 and Table 2, Figure 3 and Figure 4). Steven’s power relationship modeling performed consistently better than logarithmic relationship modeling for resting tremor data, while differences for postural tremor were less consistent. Logarithmic regression particularly struggled with correctly predicting higher tremor scores (Supplementary Figures S2 and S3).

### 3.3. Second Rater Data

Additionally, n = 100 trials of each resting and postural tremor were rated by a second rater. Weighted Kappa between raters was κw = 0.869 for resting tremor and κw = 0.719 for postural tremor. Accelerometry predictions of the second rater’s ratings performed slightly worse than for our validation data, but once again, acceleration-based metrics slightly outperformed frequency-based metrics. Mean Acceleration performed best for Resting Tremor and FingerACC (κw = 0.738) and Postural Tremor and WristACC (κw = 0.704). Mean Envelope performed best for Resting Tremor and WristACC (κw = 0.692) and Postural Tremor and FingerACC (κw = 0.725). Full results for the second rater can be found in Supplementary Tables S1 and S2.

### 3.4. Comparison between ET and PD

**Figure 5:**
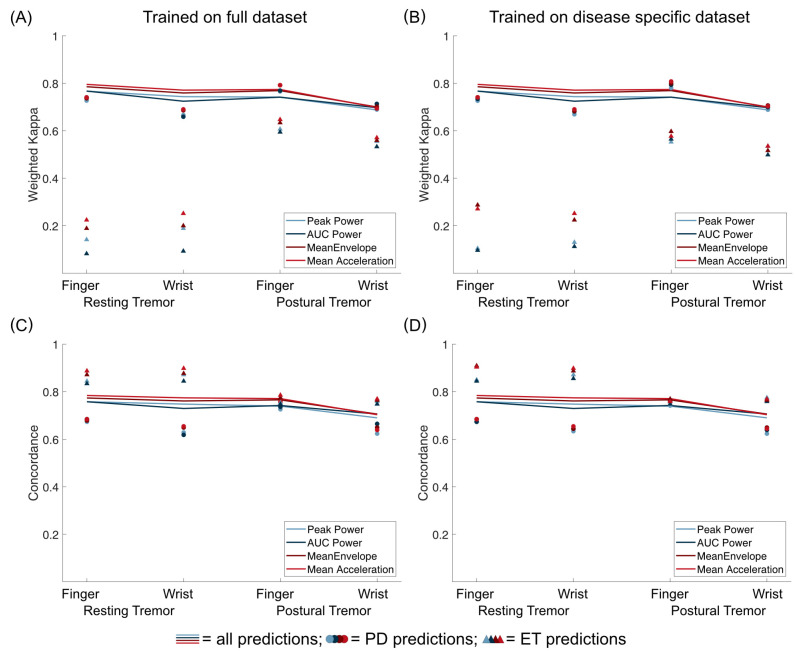
Comparison of predictions for all validation data (lines), only data from PD subjects (circles), and only data from ET subjects (triangles). Weighted Kappa coefficients are shown in (**A**,**B**), while concordance between predictions and rater is shown in C and D. On the left (**A**,**C**), results are shown for models trained on the full training dataset. On the right (**B**,**D**), results are shown when only subjects with the same disease were used for training the models.

To compare predictions of ET and PD tremor, we first used our models, which were trained on the full *training dataset* to predict only subjects with ET or PD in our *validation dataset* (Figure 5). Notably, weighted Kappa values were lower when predicting ET, especially for resting tremor predictions. Concordance between predictions and rater, however, was similar or even higher for ET. This discrepancy can be mainly attributed to very unbalanced data for the ET group, especially for resting tremor, where no scores higher than two were observed in the validation group of ET subjects. Secondly, we trained our models once more, this time only taking into account subjects with ET or PD from the *training dataset* to predict the data from the respective subjects with the same disorder in the *validation dataset*. As highlighted in Figure 5, this only impacted prediction results to a very minor degree, demonstrating that pooling accelerometry data from ET and PD subjects are feasible. Importantly, Mean Acceleration also proved to be the best metric in these post hoc analyses.

## 4. Discussion

This study highlights the value of utilizing accelerometry to assess tremor amplitude for both resting and postural tremors in PD and ET employing finger- and wrist-mounted devices. We demonstrate that different tremor metrics allow a reliable prediction of tremor severity in out-of-sample data. Simple metrics based on acceleration within the band of possible tremor frequencies (3 to 12 Hz) seem to provide optimal results for most scenarios. The mean error of predicting tremor ratings was far below the rating scale resolution, and predicted tremor ratings based on the proposed models only very rarely deviated by more than one point from clinical ratings. Interrater reliability between accelerometry-based tremor assessments and clinical ratings was within the range that is usually observed between clinical raters, both in this trial and in other studies [6,8]. For resting tremor, wrist-worn accelerometry provides comparable results to finger-mounted accelerometry. For postural tremor, however, measuring at more distal locations seems slightly more accurate than on the wrist.

While accelerometry allows for a reliable approximation of clinical ratings of tremor amplitude, it is not hampered by the many limitations of clinical ratings and clinical rating scales. As in our study, clinical ratings are often employed as a fictitious “gold standard” against which accelerometry is validated. For such validation purposes, reducing gradual tremor measurements to arbitrarily defined 5-point ordinal scales is necessary. When using accelerometry in clinical or scientific applications, however, it is, of course, much more meaningful to use gradual metrics of tremor, which allow an accurate assessment of tremor severity. Furthermore, accelerometry has the advantage of being rater-independent, technically reliable, and usually free of technical artifacts. Additionally, it can be used for continuous monitoring with minimum interference with daily living, especially when worn on the wrist. We thus see many potential uses of accelerometry. First of all, we think that accelerometry should be used in most, if not all, scientific research of tremor since it provides objective, fine-grained, and gradual outcomes, which are much more suitable for statistical analyses than nominal or ordinal scales. Second, we think that including such metrics in the clinical workflow is meaningful, especially when different clinicians are involved in a tremor patient’s treatment over the course of a disease, to address the problem of interrater reliability of clinical scales. Third, task-based accelerometry for tremor may be helpful in telemedicine settings and for at-home monitoring of tremor symptoms when no experienced rater is present. Lastly, we see the special importance of accelerometry in DBS care since DBS allows subtle modulation of tremor severity not detectable by conventional clinical scales.

Several previous studies investigated accelerometry to assess tremors, employing various approaches and metrics. Unfortunately, many of these trials were limited to small datasets [12,15,16,32,33], and only very few studies investigated their approaches in an independent validation dataset [20]. The dataset we provide with this study provides accelerometric data with various tremor intensities and includes expert clinical rating, which offers the possibility to re-evaluate, validate, and possibly improve already established approaches. Furthermore, we propose standardizing measurement units and axes to facilitate the generalization of approaches to different devices and recordings.

Our study has several limitations. First, the model has been established based on ratings from one rater. Still, we demonstrated substantial interrater reliability when assessing a subset of our data by a second rater. Future investigations may clarify whether a more heterogeneous training dataset, assessed by a larger number of raters, can lead to superior prediction models. Second, our dataset was not balanced regarding tremor severity, and, as in other studies, there were fewer trials with severe tremor than with no or less severe tremor [15,20]. This might have influenced the training of the logarithmic relationship models, which often misclassified severe, four-point-rating tremors. While severe tremor is less common, it needs to be discussed whether training of approaches would be more meaningful in more balanced datasets. Third, we only investigated a few simple metrics to analyze our accelerometry data. While these metrics performed well compared to previous studies [12,15,20,33], more complex metrics or models combining multiple parameters might further improve results. We welcome anyone to investigate such approaches within our released dataset. We deliberately chose to limit our devices to triaxial accelerometry since accelerometry is widely available and also present in a variety of consumer electronics. We utilized accelerometry devices with previous applications for tremor evaluation. However, other accelerometry devices might be even more accurate [34], and other technical solutions like, e.g., gyroscopes, might be equally or possibly even better suited to classify tremor. Lastly, it is important to highlight that this study used task-based tremor assessments in a clinical setting and thus did not investigate tremor severity in real life and during activities of daily living. While others have used such approaches—and even gained some regulatory approval—to continuously assess tremor in ambulatory settings [20], we would argue that such implementations should be rigorously scrutinized [35]. Real-life usage of wearable accelerometry is different from task-based usage so that real-life usage requires additional validation, e.g., by detailed movement diaries or, even better, objective in-person or video monitoring.

## 5. Conclusions

With a large-scale dataset, we demonstrated that automatic assessments of upper limb tremor severity with accelerometry at the finger or wrist are feasible for both ET and PD tremors—and that even simple computational models offer reliable and precise approximations of established tremor scales. Accelerometry offers multiple advantages over clinical rating scales, but the results need to be validated using out-of-sample data. Furthermore, the detailed release of accelerometry data, model parameters, algorithms, and software—as undertaken in this study—is warranted to allow for external cross-validation and improvement of existing methods. We hope our analyses and data will help to further promote the application of accelerometry in the research and clinical care of tremor.

## Figures and Tables

**Table 1:** Model parameters and prediction accuracies for logarithmic relationships models. The best tremor metric for each tremor type and accelerometry location is highlighted in grey.

Tremortype	Location	Metric	TR = a × ln(T) + b		RMSE	Weighted Kappa	Concordance	1 Pt Deviation	>1 Pt Deviation
			a	b					
Resting Tremor	Finger	Peak Power	0.223	−0.257	0.528	0.708	68.1%	31.4%	0.5%
		AUC Power	0.244	2.601	0.527	0.72	69.9%	29.8%	0.3%
		Mean Envelope	0.532	1.958	0.51	0.724	70.2%	29.6%	0.3%
		Mean Acceleration	0.502	2.68	0.492	0.734	70.7%	29%	0.3%
	Wrist	Peak Power	0.258	3.368	0.537	0.707	71.3%	27.1%	1.6%
		AUC Power	0.3	3.22	0.536	0.713	71.8%	26.6%	1.6%
		Mean Envelope	0.671	2.456	0.518	0.724	72.6%	26.1%	1.3%
		Mean Acceleration	0.631	3.321	0.489	0.743	74.7%	23.9%	1.3%
Postural Tremor	Finger	Peak Power	0.281	3.084	0.515	0.721	72%	27.4%	0.5%
		AUC Power	0.318	2.852	0.511	0.76	76%	23.2%	0.8%
		Mean Envelope	0.701	1.997	0.502	0.769	76.5%	23%	0.5%
		Mean Acceleration	0.655	2.932	0.485	0.772	76.8%	22.7%	0.5%
	Wrist	Peak Power	0.296	3.707	0.581	0.697	70.3%	27.4%	2.4%
		AUC Power	0.363	3.7	0.581	0.703	70.8%	26.1%	3.2%
		Mean Envelope	0.819	2.802	0.569	0.722	72.6%	24.2%	3.2%
		Mean Acceleration	0.761	3.812	0.565	0.728	73.2%	23.7%	3.2%

**Table 2:** Model parameters and prediction accuracies for Steven’s power relationships models. The best tremor metric for each tremor type and accelerometry location is highlighted in grey.

Tremortype	Location	Metric	TR = a × T^c^ + b			RMSE	Weighted Kappa	Concordance	1 Pt Deviation	>1 Pt Deviation
			a	b	c					
Resting Tremor	Finger	Peak Power	0.757	−0.83	0.13	0.448	0.767	75.7%	23.7%	0.5%
		AUC Power	3.963	−0.872	0.136	0.461	0.767	75.7%	23.7%	0.5%
		Mean Envelope	2.985	−1.106	0.257	0.461	0.752	73.9%	25.9%	0.3%
		Mean Acceleration	4.315	−1.244	0.226	0.438	0.795	78.4%	21.4%	0.3%
	Wrist	Peak Power	1.586	−1.669	0.098	0.505	0.744	74.7%	23.9%	1.3%
		AUC Power	6.919	−3.28	0.069	0.52	0.724	72.9%	25.5%	1.6%
		Mean Envelope	6.793	−4.296	0.123	0.51	0.732	73.2%	25.5%	1.3%
		Mean Acceleration	9.522	−5.949	0.089	0.483	0.771	77.4%	21.3%	1.3%
Postural Tremor	Finger	Peak Power	2.398	−2.872	0.071	0.505	0.742	73.9%	25.6%	0.5%
		AUC Power	8.164	−5.153	0.05	0.509	0.741	74.1%	25.1%	0.8%
		Mean Envelope	7.471	−5.496	0.105	0.503	0.751	74.7%	24.8%	0.5%
		Mean Acceleration	11.214	−8.17	0.07	0.486	0.774	77%	22.4%	0.5%
	Wrist	Peak Power	1.346	−1.492	0.119	0.592	0.688	68.9%	27.9%	3.2%
		AUC Power	10.157	−6.048	0.05	0.585	0.697	70.5%	25.5%	3.9%
		Mean Envelope	12.235	−9.358	0.077	0.575	0.714	71.3%	25.5%	3.2%
		Mean Acceleration	11.378	−7.186	0.091	0.573	0.699	70.3%	25.8%	3.9%

## Data Availability

All scripts and data are available under the creative commons license CC BY-SA 4.0 and can be found at https://dx.doi.org/10.17605/OSF.IO/MP5HA (accessed on 17 September 2023).

## Supplementary Materials

The following supporting information can be downloaded at: https://www.mdpi.com/article/10.3390/s23208621/s1, Figure S1: Schematic overview of the employed devices, the recording locations, and the coordinate system used in this study. Figure S2: Comparison of accelerometry metrics and clinical scores for resting tremor for logarithmic relationship; Figure S3: Comparison of accelerometry metrics and clinical scores for postural tremor for logarithmic relationship; Table S1: Model parameters and prediction accuracies for logarithmic relationship models, rated by a second rater; Table S2: Model parameters and prediction accuracies for Steven’s power relationships models, rated by a second rater.

## References

[1] Fahn S., Tolosa E., Marin C. (1993). Clinical Rating Scale for Tremor. Parkinson’s Disease and Movement Disorders.

[2] Elble R.J. (2016). The Essential Tremor Rating Assessment Scale. J. Neurol..

[3] Goetz C.G., Tilley B.C., Shaftman S.R., Stebbins G.T., Fahn S., Martinez-Martin P., Poewe W., Sampaio C., Stern M.B., Dodel R. (2008). Movement Disorder Society-Sponsored Revision of the Unified Parkinson’s Disease Rating Scale (MDS-UPDRS): Scale Presentation and Clinimetric Testing Results. Mov. Disord..

[4] Elble R.J., Ondo W. (2022). Tremor Rating Scales and Laboratory Tools for Assessing Tremor. J. Neurol. Sci..

[5] Elble R.J., Pullman S.L., Matsumoto J.Y., Raethjen J., Deuschl G., Tintner R. (2006). Tremor Amplitude Is Logarithmically Related to 4- and 5-Point Tremor Rating Scales. Brain.

[6] Stacy M.A., Elble R.J., Ondo W.G., Wu S.-C., Hulihan J., Group T.S. (2007). Assessment of interrater and intrarater reliability of the Fahn–Tolosa–Marin Tremor Rating Scale in essential tremor. Mov. Disord..

[7] Richards M., Marder K., Cote L., Mayeux R. (1994). Interrater Reliability of the Unified Parkinson’s Disease Rating Scale Motor Examination. Mov. Disord..

[8] Post B., Merkus M.P., de Bie R.M.A., de Haan R.J., Speelman J.D. (2005). Unified Parkinson’s Disease Rating Scale Motor Examination: Are Ratings of Nurses, Residents in Neurology, and Movement Disorders Specialists Interchangeable?. Mov. Disord..

[9] Bain P.G., Findley L.J., Atchison P., Behari M., Vidailhet M., Gresty M., Rothwell J.C., Thompson P.D., Marsden C.D. (1993). Assessing Tremor Severity. J. Neurol. Neurosurg. Psychiatry.

[10] Evers L.J.W., Krijthe J.H., Meinders M.J., Bloem B.R., Heskes T.M. (2019). Measuring Parkinson’s Disease over Time: The Real-World within-Subject Reliability of the MDS-UPDRS. Mov. Disord..

[11] Grimaldi G., Manto M. (2010). Neurological Tremor: Sensors, Signal Processing and Emerging Applications. Sensors.

[12] Gauthier-Lafreniere E., Aljassar M., Rymar V.V., Milton J., Sadikot A.F. (2022). A Standardized Accelerometry Method for Characterizing Tremor: Application and Validation in an Ageing Population with Postural and Action Tremor. Front. Neuroinform..

[13] Elble R.J., McNames J. (2016). Using Portable Transducers to Measure Tremor Severity. Tremor Hyperkinetic Mov..

[14] Lukšys D., Jonaitis G., Griškevičius J. (2018). Quantitative Analysis of Parkinsonian Tremor in a Clinical Setting Using Inertial Measurement Units. Park. Dis..

[15] Smid A., Elting J.W.J., van Dijk J.M.C., Otten B., Oterdoom D.L.M., Tamasi K., Heida T., van Laar T., Drost G. (2022). Intraoperative Quantification of MDS-UPDRS Tremor Measurements Using 3D Accelerometry: A Pilot Study. J. Clin. Med..

[16] Zach H., Dirkx M.F., Roth D., Pasman J.W., Bloem B.R., Helmich R.C. (2020). Dopamine-Responsive and Dopamine-Resistant Resting Tremor in Parkinson Disease. Neurology.

[17] Paschen S., Forstenpointner J., Becktepe J., Heinzel S., Hellriegel H., Witt K., Helmers A.-K., Deuschl G. (2019). Long-Term Efficacy of Deep Brain Stimulation for Essential Tremor: An Observer-Blinded Study. Neurology.

[18] Smid A., Oterdoom D.L.M., Pauwels R.W.J., Tamasi K., Elting J.W.J., Absalom A.R., Van Laar T., Van Dijk J.M.C., Drost G. (2023). The Relevance of Intraoperative Clinical and Accelerometric Measurements for Thalamotomy Outcome. J. Clin. Med..

[19] San-Segundo R., Zhang A., Cebulla A., Panev S., Tabor G., Stebbins K., Massa R.E., Whitford A., de la Torre F., Hodgins J. (2020). Parkinson’s Disease Tremor Detection in the Wild Using Wearable Accelerometers. Sensors.

[20] Powers R., Etezadi-Amoli M., Arnold E.M., Kianian S., Mance I., Gibiansky M., Trietsch D., Alvarado A.S., Kretlow J.D., Herrington T.M. (2021). Smartwatch Inertial Sensors Continuously Monitor Real-World Motor Fluctuations in Parkinson’s Disease. Sci. Transl. Med..

[21] Sahin G., Halje P., Uzun S., Jakobsson A., Petersson P. (2022). Tremor Evaluation Using Smartphone Accelerometry in Standardized Settings. Front. Neurosci..

[22] Kostikis N., Hristu-Varsakelis D., Arnaoutoglou M., Kotsavasiloglou C. (2015). A Smartphone-Based Tool for Assessing Parkinsonian Hand Tremor. IEEE J. Biomed. Health Inform..

[23] Fujikawa J., Morigaki R., Yamamoto N., Nakanishi H., Oda T., Izumi Y., Takagi Y. (2022). Diagnosis and Treatment of Tremor in Parkinson’s Disease Using Mechanical Devices. Life.

[24] Shah A., Coste J., Lemaire J.-J., Taub E., Schüpbach W.M.M., Pollo C., Schkommodau E., Guzman R., Hemm-Ode S. (2017). Intraoperative Acceleration Measurements to Quantify Improvement in Tremor during Deep Brain Stimulation Surgery. Med. Biol. Eng. Comput..

[25] Mcgurrin P., Mcnames J., Wu T., Hallett M., Haubenberger D. (2021). Quantifying Tremor in Essential Tremor Using Inertial Sensors—Validation of an Algorithm. IEEE J. Transl. Eng. Health Med..

[26] Brainard D.H. (1997). The Psychophysics Toolbox. Spat. Vis..

[27] Pelli D.G. (1997). The VideoToolbox Software for Visual Psychophysics: Transforming Numbers into Movies. Spat. Vis..

[28] Vergara-Diaz G., Daneault J.-F., Parisi F., Admati C., Alfonso C., Bertoli M., Bonizzoni E., Carvalho G.F., Costante G., Fabara E.E. (2021). Limb and Trunk Accelerometer Data Collected with Wearable Sensors from Subjects with Parkinson’s Disease. Sci. Data.

[29] Haubenberger D., Hallett M. (2018). Essential Tremor. N. Engl. J. Med..

[30] Bhatia K.P., Bain P., Bajaj N., Elble R.J., Hallett M., Louis E.D., Raethjen J., Stamelou M., Testa C.M., Deuschl G. (2018). Consensus Statement on the Classification of Tremors. from the Task Force on Tremor of the International Parkinson and Movement Disorder Society. Mov. Disord..

[31] Portugal R.D., Svaiter B.F. (2011). Weber-Fechner Law and the Optimality of the Logarithmic Scale. Minds Mach..

[32] Delrobaei M., Memar S., Pieterman M., Stratton T.W., McIsaac K., Jog M. (2018). Towards Remote Monitoring of Parkinson’s Disease Tremor Using Wearable Motion Capture Systems. J. Neurol. Sci..

[33] Mostile G., Giuffrida J.P., Adam O.R., Davidson A., Jankovic J. (2010). Correlation between Kinesia System Assessments and Clinical Tremor Scores in Patients with Essential Tremor. Mov. Disord..

[34] Afaq S., Loh M., Kooner J., Chambers J. (2020). Evaluation of Three Accelerometer Devices for Physical Activity Measurement Amongst South Asians and Europeans. Phys. Act. Health.

[35] Bloem B.R., Post E., Hall D.A. (2023). An Apple a Day to Keep the Parkinson’s Disease Doctor Away?. Ann. Neurol..

