# Supplementary material for: Accelerometric Classification of Resting and Postural Tremor Amplitude
Source: Sensors (Basel). 2023 Oct 21;23(20):8621. doi: 10.3390/s23208621 (PMC10611060; doi:10.3390/s23208621)
Supplement: Supplementary file 1 [file sensors-23-08621-s001.zip › sensors-2644397-supplementary.pdf]

Supplementary Materials

Supplementary Material S1—Illustration of devices & recording locations

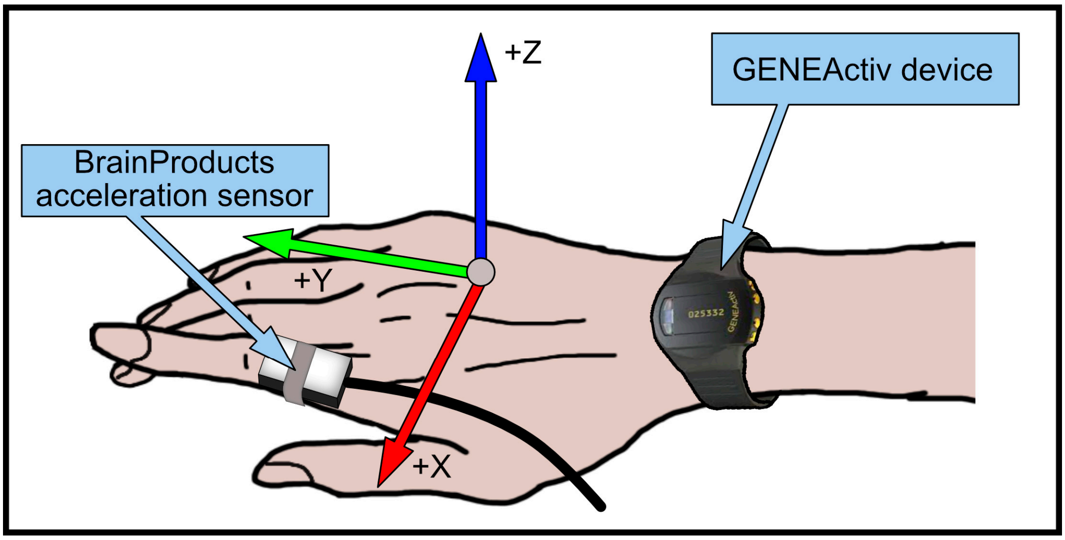

Figure S1. Schematic overview of the employed devices, the recording locations, and the coordinate system used in this study.

Supplementary Material S2—Logarithmic relationships

Analogous to Figures S2 & S3 of the manuscript, which provided results for Steven's power relationships, we here provide results for modeling and classifying the logarithmic relationships for resting and postural tremors.

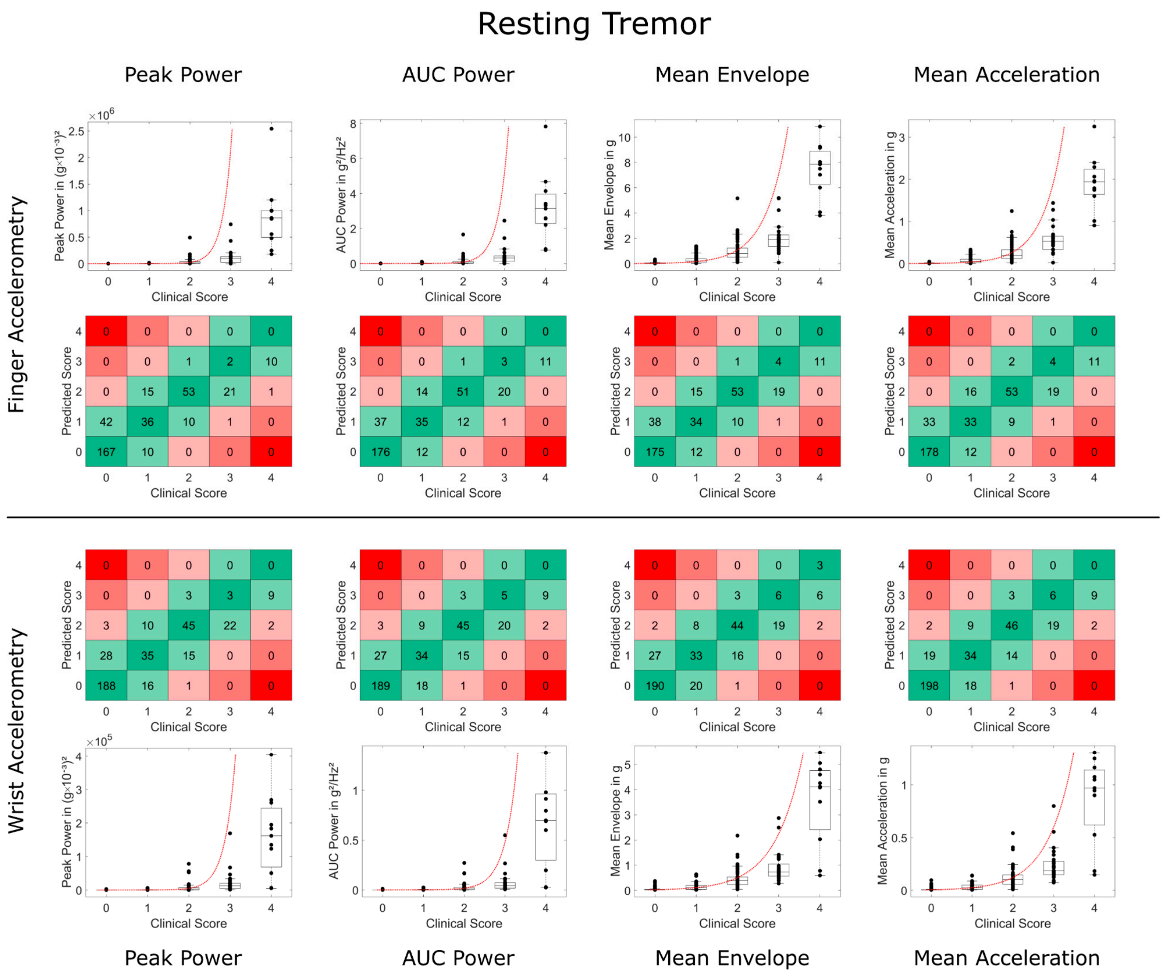

Figure S2. Comparison of accelerometry metrics and clinical scores for resting tremor for logarithmic relationship. Data on finger and wrist accelerometry are shown in the two upper and lower rows, respectively. Boxplots and black data points illustrate the validation data and the logarithmic model trained on the training data (in red). Square plots depict deviations between the predicted tremor score and clinical tremor rating of more than 1 point in red (2 points deviation in light red, 3 and 4 points deviation in dark red). High agreement (no deviation) is highlighted in dark green, deviation of only 1 point in light green.

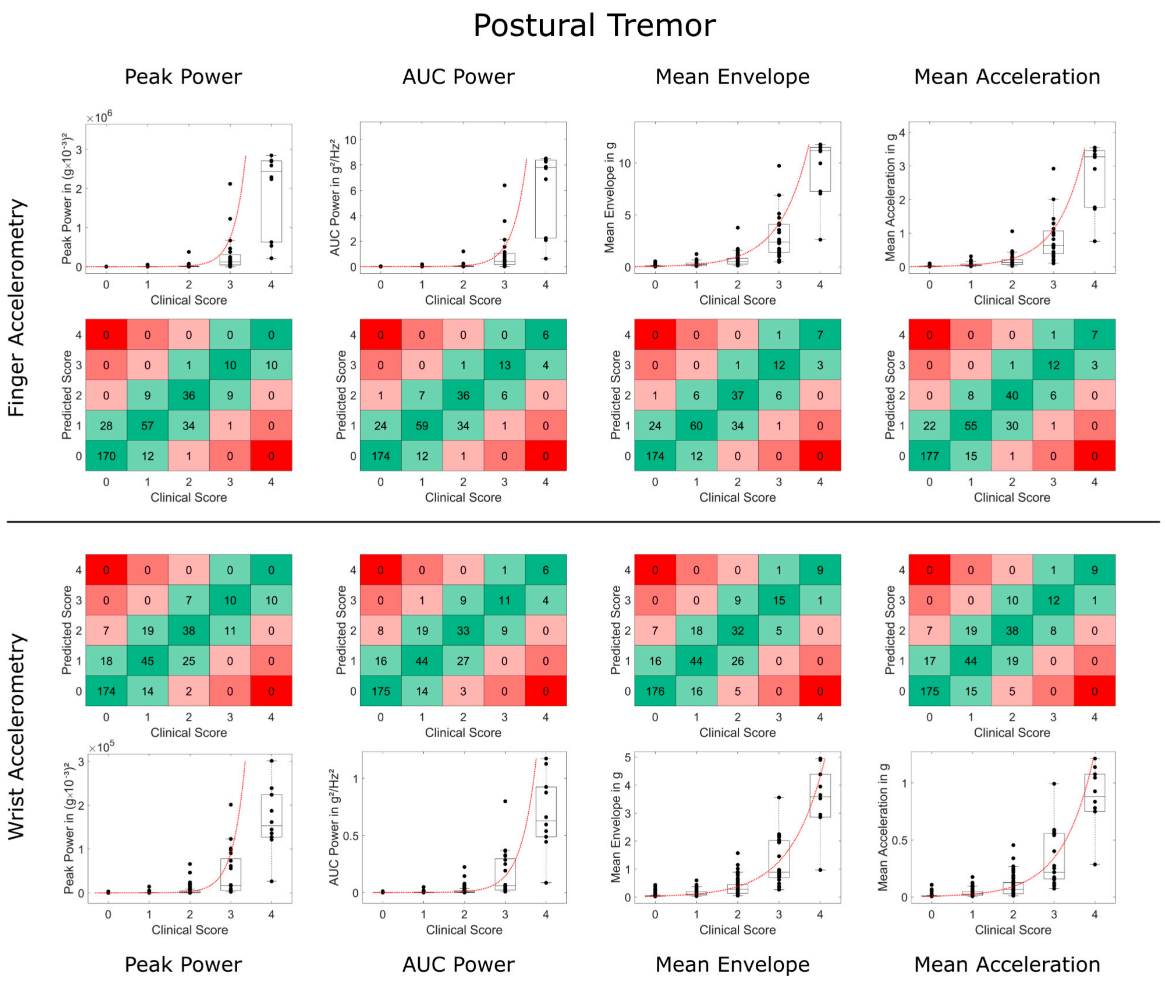

Figure S3: Comparison of accelerometry metrics and clinical scores for postural tremor for logarithmic relationship. Data on finger and wrist accelerometry are shown in the two upper and lower rows, respectively. Boxplots and black data points depict the validation data and the logarithmic model trained on the training data (in red). Square plots show deviations between the predicted tremor score and clinical tremor rating of more than 1 point in red (2 points deviation in light red, 3 and 4 points deviation in dark red). High agreement (no deviation) is highlighted in dark green, deviation of only 1 point in light green.

Supplementary Material S3—Classification Results for Second Rater

Analogous to Tables S1 & S2 of the main manuscript, these tables provide the classification results when comparing our trained models with the data rated by the second rater (GB).

Table S1: Model parameters and prediction accuracies for logarithmic relationship models, rated by a second rater. The best tremor metrics for each tremor type and accelerometry location are highlighted in grey. AUC Power = area under the power spectral density curve, RMSE = root mean standard error.

Tremortype	Location	Metric	TR = a × ln(T) + b		RMSE	Weighted Kappa	Concordance	1 Pt Deviation	>1 Pt Deviation
			a	b					
Resting Tremor	Finger	Peak Power	0.223	-0.257	0.791	0.629	54.5 %	38.4 %	7.1 %
		AUC Power	0.244	2.601	0.781	0.656	56.6 %	38.4 %	5.1 %
		Mean Envelope	0.532	1.958	0.757	0.671	58.6 %	36.4 %	5.1 %
		Mean Acceleration	0.502	2.68	0.739	0.669	56.6 %	39.4 %	4 %
	Wrist	Peak Power	0.258	3.368	0.812	0.634	55 %	38 %	7 %
		AUC Power	0.3	3.22	0.782	0.636	54 %	40 %	6 %
		Mean Envelope	0.671	2.456	0.76	0.675	56 %	41 %	3 %
		Mean Acceleration	0.631	3.321	0.749	0.652	54 %	42 %	4 %
Postural Tremor	Finger	Peak Power	0.281	3.084	0.699	0.654	59.6 %	36.4 %	4 %
		AUC Power	0.318	2.852	0.658	0.716	64.6 %	33.3 %	2 %
		Mean Envelope	0.701	1.997	0.637	0.728	64.6 %	34.3 %	1 %
		Mean Acceleration	0.655	2.932	0.648	0.719	65.7 %	31.3 %	3 %
	Wrist	Peak Power	0.296	3.707	0.749	0.643	58 %	37 %	5 %
		AUC Power	0.363	3.7	0.692	0.676	59 %	38 %	3 %
		Mean Envelope	0.819	2.802	0.681	0.702	62 %	35 %	3 %
		Mean Acceleration	0.761	3.812	0.698	0.693	62 %	35 %	3 %

Table S2: Model parameters and prediction accuracies for Steven's power relationships models, rated by a second rater. The best tremor metrics for each tremor type and accelerometry location are highlighted in grey. AUC Power = area under the power spectral density curve, RMSE = root mean standard error.

Tremortype	Location	Metric	TR = a × T^c + b			RMSE	Weighted Kappa	Concordance	1 Pt Deviation	>1 Pt Deviation
			a	b	c					
Resting Tremor	Finger	Peak Power	0.757	-0.83	0.13	0.713	0.724	64.6 %	31.3 %	4 %
		AUC Power	3.963	-0.872	0.136	0.721	0.731	65.7 %	30.3 %	4 %
		Mean Envelope	2.985	-1.106	0.257	0.709	0.717	62.6 %	34.3 %	3 %
		Mean Acceleration	4.315	-1.244	0.226	0.691	0.738	65.7 %	31.3 %	3 %
	Wrist	Peak Power	1.586	-1.669	0.098	0.782	0.654	55 %	40 %	5 %
		AUC Power	6.919	-3.28	0.069	0.774	0.654	54 %	42 %	4 %
		Mean Envelope	6.793	-4.296	0.123	0.759	0.692	59 %	38 %	3 %
		Mean Acceleration	9.522	-5.949	0.089	0.747	0.669	56 %	40 %	4 %
Postural Tremor	Finger	Peak Power	2.398	-2.872	0.071	0.666	0.681	60.6 %	36.4 %	3 %
		AUC Power	8.164	-5.153	0.05	0.642	0.694	62.6 %	34.3 %	3 %
		Mean Envelope	7.471	-5.496	0.105	0.625	0.725	63.6 %	35.4 %	1 %

	Mean Acceleration	11.214	-8.17	0.07	0.639	0.705	62.6 %	35.4 %	2 %
Wrist	Peak Power	1.346	-1.492	0.119	0.708	0.665	59 %	36 %	5 %
	AUC Power	10.157	-6.048	0.05	0.683	0.704	62 %	35 %	3 %
	Mean Envelope	12.235	-9.358	0.077	0.678	0.695	62 %	35 %	3 %
	Mean Acceleration	11.378	-7.186	0.091	0.694	0.704	63 %	34 %	3 %
